# Mr. Barlow's Letter to Mr. Ward, on Frictions with Opium

**Published:** 1799-09

**Authors:** M. Ward

**Affiliations:** Manchester


### *To the Editors of the Medical and Phyfical Journal.*

GENTLEMEN,

THE following communication I have received from Mr. BARLOW, Surgeon, at Bolton.

As

As Mr. B. alludes to a ſubject which has appeared in the Medical and Phyſical Journal, and as the caſe he relates is of conſiderable practical importance, I ſhall be glad if you will give it a place in the next Number of your uſeful publication; and am,

Your very humble Servant,

MANCHESTER,

*Auguſt* 11, 1799.

M. WARD.

"Agreeably to your requeſt I ſend you the annexed caſe, which you are at liberty to publiſh or not, as you may think proper.

"I did not keep a journal of the caſe, which will account for my not mentioning every minute particular.

I am, Sir,
Yours, &c.

BOLTON, *Auguſt* 10, 1799.

J. BARLOW.

"Eſther Knowles, aged 49, was ſuddenly attacked about the middle of laſt June, with a ſhooting pain on the inſide of her right thumb, near the ſecond joint. The pain did not continue long; but about the eighth day it returned, and a purple bliſter appeared upon the part, about the ſize of a ſmall pea.

"Not thinking it of much conſequence, ſhe allowed a week to elapſe before ſhe conſulted me. The whole arm was ſwelled, particularly along the courſe of the lymphatics, as high as the axilla, where there was a tumour of the glands. The ulcer, which was about the breadth of a ſhilling, continued ſpreading, and had a gangrenous appearance: the pain was exceſſive, and as ſhe expreſſed herſelf, ſhot from her thumb to her heart.

"I ordered leeches to be applied to her arm, and alſo a fomentation of poppy-heads, and a common poultice, morning and evening.

"On the following day, the inflamation was conſiderably abated; but the pain continued, and the ulcer looked no better.

"I gave her opium, both in the ſolid and liquid form, with as much bark and wine as her ſtomach could bear, which ſhe took a week without much abatement of the pain; the ulcer ſtill ſpreading. She was then ſo weak and faint, that ſhe could not come to me to be dreſſed, as uſual.

"I viſited her the next morning, and found her in bed, her ſtomach rejecting every thing ſhe took, whether food or medicine; and if ſhe raiſed her head, ſhe fainted.

"Having lately read your paper in the Medical and Phyſical Journal, on the effects of opium applied externally, ſo as to be abſorbed by the lymphatics; phatics; and thinking that plan of treatment worthy of being tried in the present case, I directed a liniment, with six drachms of tincture of opium and an equal quantity of olive oil, combined by the yolk of an egg; the whole of which was rubbed on the inside of the patient's legs and thighs, at three times in the course of the day.

"The next morning she was easier, and the ulcer did not appear to have spread.

"The frictions were continued five days longer, with an ounce of the tincture of opium each day; during which time the pain left her, and the ulcer looked better every day.

"As she had by this time tolerably recovered her appetite and strength, I only saw her occasionally, and gave her, now and then, an opium pill at at night.

"The pulse from the time I saw her, was low, and not remarkably quick."

---

